# Who labels best? Radiologists, rules, or large language models for CT reports on pulmonary embolism

**DOI:** 10.1186/s41747-026-00738-7

**Published:** 2026-05-27

**Authors:** Matthias A. Fink, Arved Bischoff, Edem Atsiatorme, Alexander Kremer, Jonas Kroschke, Martin Moll, Patrick Stein, Veronika Riebl, Timo Leichenich, Hans-Ulrich Kauczor, Kai Schlamp

**Affiliations:** 1 Clinic for Diagnostic and Interventional Radiology, University Hospital Heidelberg, Heidelberg, Germany; 2 Translational Lung Research Center Heidelberg, member of the German Center for Lung Research, Heidelberg, Germany; 3 Diagnostic and Interventional Radiology with Nuclear Medicine, Heidelberg Thoracic Clinic, University of Heidelberg, Heidelberg, Germany

**Keywords:** Data mining, Large language models, Natural language processing, Pulmonary embolism, Structured reporting

## Abstract

**Objective:**

To compare open-weight and proprietary large language models (LLMs), a rule-based extractor (RBE) and radiologists for labelling pulmonary embolism CT reports, and to test whether a hybrid RBE–LLM workflow improves labelling performance.

**Materials and methods:**

This single-centre retrospective study included structured CT reports from October 2021 to March 2025. Three labelling pipelines were evaluated: an RBE; a model-agnostic LLM extractor (18 open-weight, four GPT-4 variants); and a hybrid pipeline routing only RBE failures to an LLM. Ground truth was defined at the report-text level by deterministic schema matching for initially RBE-valid fields and blinded adjudication of RBE-invalid fields by two attending radiologists. Eight radiologists provided a human baseline. Outcomes included F1 scores, accuracy, LLM-based salvage of RBE failures, and labelling time.

**Results:**

In total, 2,923 reports from 2,923 patients (mean age 66 ± 17 years; 1,465 women) were included. Falcon3-10b and GPT-4.1-mini achieved similar item-level performance (F1 0.98 [95% CI, 0.97–0.98] for both; *p* = 0.70) and both exceeded the RBE (F1 0.81 [95% CI, 0.80–0.82]; *p* < 0.001). Salvage of RBE failures was comparable between open-weight and proprietary models (88.1% *vs* 91.9%; *p* = 0.12). The hybrid RBE–LLM workflow achieved 99.8% accuracy and F1 0.99 (0.98–0.99), exceeding both the RBE and pooled radiologists (F1 0.92 [95% CI, 0.90–0.93]; all *p* < 0.001).

**Conclusion:**

Schema-constrained open-weight and proprietary LLMs exceeded rule-based extraction and, at the upper end of performance, matched a pooled radiologist label-transfer baseline. A rules-first, targeted LLM workflow enabled near-perfect extraction from finalised structured pulmonary embolism CT reports.

**Relevance statement:**

A rules-first LLM workflow can automate high-fidelity extraction of structured CT findings from finalised radiology reports, enabling scalable, auditable, and more consistent cohort curation for clinical research, registries, and quality improvement.

**Key Points:**

- A hybrid rules-first workflow combining a rule-based extractor (RBE) with targeted large language model (LLM) salvage achieved the highest overall performance for labelling of pulmonary embolism CT reports (F1, 0.99; accuracy, 99.8%).
- The top standalone open-weight and proprietary LLMs (Falcon3-10b and GPT-4.1-mini) both exceeded the RBE and, at the upper end of performance, matched a pooled radiologist label-transfer baseline.
- The hybrid workflow reduced cohort-curation time from 32.2 h for radiologists to 1.0 h while reducing LLM calls by 85.6%, because the LLM was only triggered for rule-failed fields.

**Graphical Abstract:**

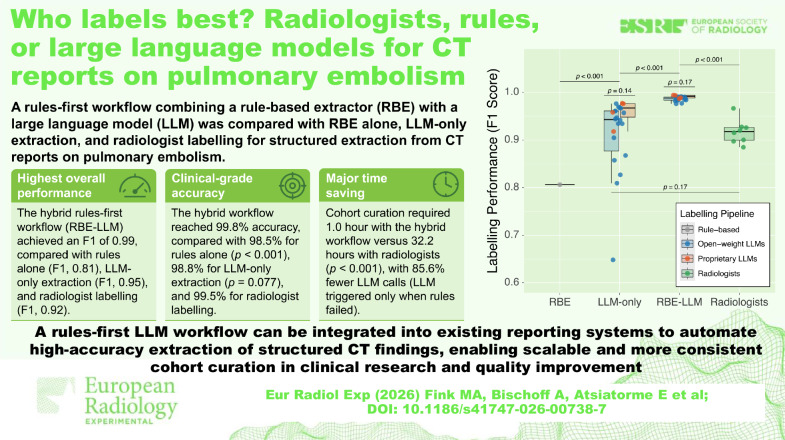

## Background

Programmatically derived labels from radiology reports are increasingly used to build imaging cohorts, train artificial intelligence models, and monitor quality of care [1]. In many institutions, the acquisition of such labels is still performed through manual chart review or by employing rule-based natural language processing techniques on radiology reports [2, 3]. These methods can be both labour-intensive and subject to variability among readers, across sites, and across report styles [4]. As datasets increase in size, the time and cost required for large-scale manual annotation become prohibitive, creating a practical need for robust, automated labelling strategies.

Large language models (LLMs) have been shown to be capable of extracting structured information from clinical text with minimal task-specific training and have recently been applied to radiology reporting tasks such as entity extraction, impression summarisation, and report classification [5–8]. In contrast to manually crafted rules, LLMs have the capacity to manage heterogeneous phrasing, uncertainty, and negation, and can, in principle, be reused across institutions and languages [9]. However, routine deployment in clinical environments is constrained by privacy and governance concerns, dependency on proprietary cloud application programming interfaces (APIs), and computational cost [10]. The question of whether LLMs should primarily complement or replace existing rule-based pipelines in daily practice remains unresolved.

Pulmonary embolism (PE) CT templates are widely used to standardise reporting of clot burden, right heart strain, and perfusion deficits, and are increasingly adopted in European centres [2, 11, 12]. Accurate extraction of these fields is essential for downstream research, registry building and decision-support tools, because even minor systematic errors in PE status or severity can introduce bias in outcome analyses. Conversely, minor deviations from the prescribed template, such as free-text additions, alternative wording, or incomplete lists, may break exact-match rules and necessitate manual correction, particularly in structured reports that combine predefined options with free text.

Therefore, this study had three aims: first, to compare the labelling performance of open-weight and proprietary LLMs with a rule-based extractor (RBE) for information retrieval from structured CT reports; second, to evaluate a hybrid RBE–LLM workflow that routes only rule-based extraction failures to an LLM; and third, to benchmark the automated pipelines against a human baseline from eight radiologists.

## Materials and methods

### Study design and population

**Fig. 1:**
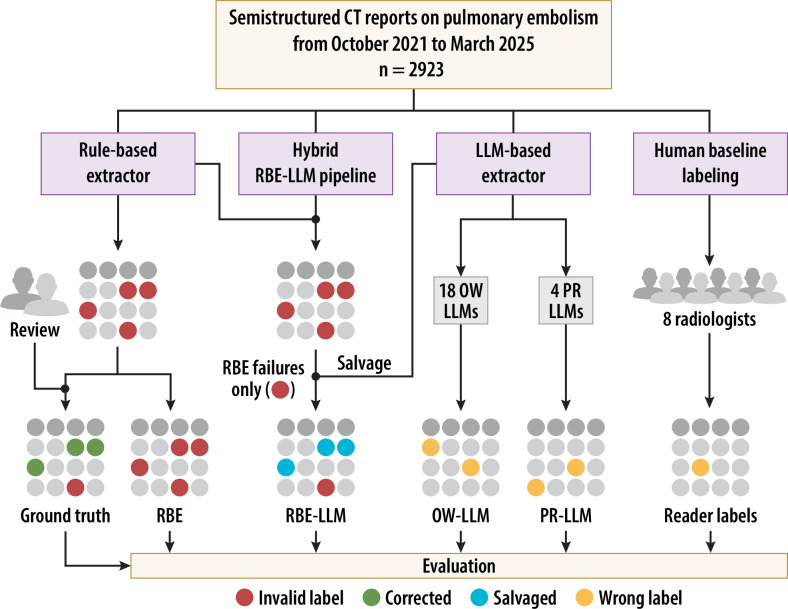
Study flow, labelling pipelines, and human baseline. Structured pulmonary angiography CT reports were processed by a rule-based extractor (RBE); a hybrid workflow that routed only fields the RBE failed to label to an LLM for targeted salvage, passing RBE-valid fields through unchanged; a model-agnostic LLM extractor querying 18 open-weight models (OW-LLM) and four proprietary GPT-4 variants (PR-LLM) with structured outputs; and an independent human baseline of eight radiologists. RBE-failed fields were adjudicated by two attending radiologists; the adjudicated RBE table defined the reference standard. PE, Pulmonary embolism

This retrospective, single-centre diagnostic accuracy study was approved by the institutional review board (S-236/2020) and was conducted in accordance with the Declaration of Helsinki. The requirement for written informed consent was waived because only de-identified data were used. Consecutive CT pulmonary angiography (CTPA) reports finalised with a structured PE template were retrieved from the radiology information system between October 2021 and March 2025. The template was implemented across 14 regional hospitals within a shared radiology service, and reports from all clinical settings (outpatient, inpatient, and emergency department) were eligible. The inclusion criterion was CTPA examinations with a report using the PE template within the study period, with no additional exclusion criteria applied. To obtain a patient-level dataset, one report per patient was retained, yielding 2,923 reports authored by 89 trainees and 49 board-certified radiologists. The primary outcomes were precision, recall and item-level accuracy, as well as F1 scores, for the RBE, standalone LLMs, the hybrid RBE–LLM workflow, and the radiologist baseline. Secondary outcomes were (1) the salvage of RBE failures by LLMs and (2) the annotation time for automated pipelines and radiologists. A separate analysis focused on resources examined the time, cost and carbon footprint of these pipelines in more detail for the same cohort [13]. Figure 1 summarises the study design.

### Reporting template and rule-based extractor

**Fig. 2:**
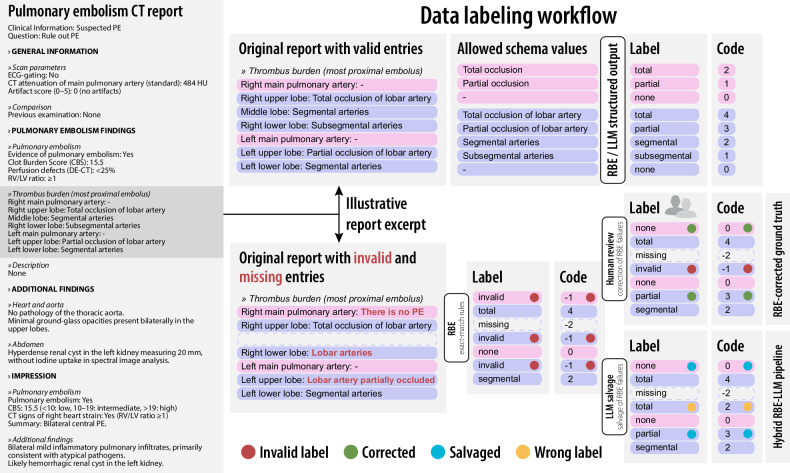
Semistructured pulmonary embolism (PE) CT report and data-labelling workflow. Left: Structured reporting template with four sections that define the extraction schema: (1) general information, (2) pulmonary embolism findings (including a nested thrombus-burden list), (3) additional findings, and (4) impression. Right, top: Allowed schema values per field and their codes used for RBE/LLM structured output. Right, middle: Report excerpt shown in a version with only valid entries (top) and a version containing invalid (out-of-schema format) and missing entries (bottom). The RBE applies exact-match rules to produce labels and codes. Human review denotes blinded manual extraction by two radiologists to correct RBE failures and form the RBE-corrected ground truth. In the hybrid RBE–LLM pipeline, only reports with RBE failures are sent to the LLM for targeted salvage with schema-constrained output. Wrong label indicates a schema-valid but incorrect value relative to the reference. CBS, Clot burden score; LLM, Large language model; RBE, Rule-based extractor; RV/LV, Right ventricle-to-left ventricle ratio; DE-CT, Dual-energy CT; HU, Hounsfield units

All reports were generated with a browser-based template, SmartReporting (SmartReporting GmbH), comprising four sections: (1) general information (*e.g*., ECG gating, pulmonary trunk attenuation, image quality); (2) PE findings, including a thrombus-burden table, clot-burden score, perfusion deficits on dual-energy CT, and right-to-left ventricle ratio; (3) additional findings; and (4) an overall impression of PE status (Fig. 2, left panel). An RBE applied exact-match rules to each predefined field and produced a standardised table. Fields were tagged as ‘missing’ when information was not reported or could not be inferred from the template. Fields were tagged as ‘invalid’ (*i.e*., RBE failures) when information was present but did not match the permitted schema, for example, non-permitted categories, mixed or free-text entries, or contradictory combinations relative to the PE status. In the hybrid pipeline, only fields classified as ‘invalid’ triggered LLM salvage; missing fields were not routed to the LLM and were excluded from performance analyses. Fields with an exact match to one permitted schema value and no failed consistency checks were treated as valid and were passed through unchanged in the hybrid workflow. Figure 2 shows the data labelling workflow, with worked examples on the right.

### Reference standard and human baseline

The reference standard was defined at the level of the report text, rather than at the level of the image diagnosis. Therefore, the study evaluated schema-constrained extraction from finalised structured reports rather than diagnostic reasoning or image-based clinical truth adjudication. The RBE generated a candidate label table for all 14 template fields. A field was classified as valid if the final report text contained an exact match for one permitted schema value, and if all field-consistency checks were satisfied. Fields were classified as missing if the information was absent from the final report text or could not be inferred from the structured template. Fields were classified as invalid if information was present, but not schema-conformant. This included *post hoc* editing, mixed entries, free-text additions, non-permitted values and contradictory cross-field combinations. Only invalid fields underwent targeted manual adjudication.

Two attending radiologists (M.A.F., 8 years of experience; K.S., 12 years) who were blinded to all LLM outputs independently reviewed all RBE-invalid fields using the finalised report text in full. They transcribed the corresponding value into the predefined schema whenever an unambiguous mapping was possible. If information remained absent, ambiguous or contradictory after full review, or could not be reliably mapped to a permitted schema token, the field was retained as invalid. Disagreements were resolved by consensus. No LLM outputs were used to define or modify the reference standard.

To estimate the residual risk of silent parser errors among initially accepted RBE-valid fields, an additional random audit of the reference standard was performed. The audit was restricted to reports with fully RBE-valid extraction and to field-level entries that (1) were not manually corrected during the initial adjudication step, (2) were retained unchanged in the final ground truth, and (3) had a corresponding independently recorded reader label available. From this pool of eligible items, we randomly selected 400 field-level entries (50 per reader), ensuring that no more than one item was sampled from each report. Each sampled item was first automatically compared against the independently recorded reader extraction. All discordant items were then manually reviewed against the source report and classified as a confirmed RBE error, a reader extraction error, a coding difference or unresolved. Appendix E3 summarises the audit workflow and item distribution.

To obtain an independent human baseline, eight radiologists annotated the entire dataset. Three were board-certified radiologists (A.B., J.K., M.M.; mean experience, 8 years) and five were residents (E.A., A.K., P.S., V.R., T.L.; mean experience, 3 years). The radiologists used a structured annotation interface with fixed dropdown options that were identical to those permitted in the reporting and LLM output schemas. Consequently, all readers, the RBE, the LLM pipelines and the final reference standard operated within the same constrained label space. Format-only mismatches due to free-text wording were therefore not possible. Each reader received a balanced, randomised assignment and was blinded to all automated outputs. A timing widget recorded the duration of each labelling task. Inter- and intra-reader variability were assessed using an overlapping subset and blinded duplicates.

### Model-agnostic LLM extractor

An LLM-agnostic extractor queried 18 open-weight and four proprietary LLMs *via* a unified, structured-output interface aligned with the CT reporting template. A zero-shot prompt provided definitions for each field and listed the permitted response options. A JavaScript Object Notation (JSON) schema enforced field names and data types across models. Where available, vendor structured-output APIs were used to improve schema adherence. Open-weight models (Falcon3-10b, Qwen2.5-72b, Llama3-3b, Qwen2.5-3b, Mistral-small-22b, Qwen3-0.6b, 1.7b, 4b, 8b, 14b, 30b, 32b and DeepSeek-R1-1.5b, 7b, 8b, 14b, 32b, 70b) were served locally *via* Ollama on a workstation equipped with an NVIDIA Quadro RTX 8000 with 48 GB of VRAM, a 12-core Intel Xeon CPU and 128 GB of RAM. Proprietary GPT-4 variants were accessed *via* the OpenAI API using dated, pinned snapshots: GPT-4o-mini (released 18 July 2024), GPT-4.1-nano and GPT-4.1-mini (both released 14 April 2025), and GPT-4.1 (released 14 April 2025). Proprietary model queries were performed on 28 May 2025 (GPT-4o-mini), 29 May 2025 (GPT-4.1-nano and GPT-4.1-mini), and 30 May 2025 (GPT-4.1). All models were queried with identical instructions, and the outputs were schema-validated and normalised into a common table. Model specifications are summarised in Appendix E1.

### Hybrid RBE–LLM pipeline

The hybrid workflow first applied the RBE. For valid labelled fields, RBE outputs were accepted without change. In the event of an RBE failure, an LLM was used to generate a schema-conformant label using the structured interface. The final hybrid table combined valid RBE entries with entries salvaged by the LLM. The hybrid outputs were then compared directly with the reference standard.

### Statistical analysis

Statistical analyses were performed by one author (M.A.F.) using R (version 4.5.1) and Python (version 3.13) software. As this was an exhaustive consecutive series of all eligible structured PE reports at our institution, an a priori power calculation was not performed. Two-sided *p*-values less than 0.05 were considered statistically significant. Differences in the baseline characteristics of the reporting items were compared using the *t*-test for continuous variables and the chi-squared test for categorical variables. For each automated data labelling workflow and LLM, item-level precision, recall, accuracy and F1 score were computed against the reference standard. For item-level precision, recall, accuracy and F1 score, 95% confidence intervals (CI) were recomputed using a report-level cluster bootstrap with 1,000 replicates. In each replicate, reports were sampled with replacement, with all evaluable template fields from each sampled report carried forward jointly to preserve within-report clustering of fields. For pooled radiologist analyses, the bootstrap unit was the annotated report instance. Confidence intervals were obtained from the percentile distribution of the bootstrap estimates. Pairwise comparisons of accuracy and F1 score between the RBE and each LLM were performed using McNemar’s test with Bonferroni correction for multiple comparisons. For the human baseline, pooled item-level performance and report-level accuracy were calculated against the reference standard. Inter-reader agreement across the 120 overlapping reports was assessed using Fleiss’ kappa statistic, and intra-reader reproducibility on 20 duplicate reports was assessed using Cohen’s kappa statistic. Reader time was summarised descriptively.

## Results

### Study sample and ground truth

CT reports from 2,923 patients (mean age, 66 ± 17 years; 1,465 women (50.1%)) were included after application of the inclusion and exclusion criteria. Each report contained 14 structured fields, yielding 40,922 data entries. Initial RBE extraction flagged 146 entries (0.4%) as missing and 769 (1.9%) as invalid. At least one invalid field was present in 420 of 2,923 reports (14.4%). Manual adjudication corrected 647 of 769 invalid entries (84.1%), leaving 122 entries (0.3% of all fields) invalid in the reference standard. Initial pre-consensus exact agreement between the two adjudicating radiologists across the 769 RBE-invalid fields was 99.2% (763/769), with six fields requiring consensus resolution. All missing entries remained missing and were excluded from performance calculations. This adjudicated RBE table served as the ground truth for all subsequent analyses.

To further validate the reference standard, we performed a random field-level audit of initially accepted RBE-valid entries. Automated comparison of 400 sampled items against independently recorded reader labels showed 399 exact matches (99.8%) and one discordant item (0.2%). Manual review of the source report attributed the discordance to a reader extraction error rather than to the RBE. Accordingly, no silent RBE parsing errors were confirmed in the audited sample (observed silent error rate: 0/400 = 0%; approximate upper 95% bound: 0.75%, calculated using the rule of three). The audit workflow and sampling distribution are shown in Appendix E3.

Patient characteristics and labelled items are summarised in Table 1. A total of 810 examinations (27.7%) showed confirmed PE, 57 (2.0%) were reported with PE suspicion, 2,027 (69.3%) showed no PE, and 26 (0.9%) were not assessable. Thrombus burden was classified as low (< 25%) in 366 reports (12.5%), intermediate (25–50%) in 143 (4.9%), and high (> 50%) in 286 (9.8%). Right heart strain (right ventricle-to-left ventricle ratio ≥ 1) was present in 639 reports (21.9%), and perfusion deficits of at least 25% were documented in 207 reports (7.1%). Pulmonary trunk attenuation was recorded in 2,922 of 2,923 reports (100%; mean, 339 ± 155 HU). Image quality was rated as no artefacts in 41.6% and minimal artefacts in 35.6% of reports; moderate-to-severe artefacts occurred in 9.0%, and 0.3% of studies were considered non-diagnostic. Among confirmed emboli, localisation was most frequently reported in the main pulmonary arteries (43.5%), followed by lobar (26.4%), segmental (20%), and subsegmental (7.5%) branches. Detailed clot-level distributions are provided in Appendix E2.

**Table 1:** Patient characteristics and labelled items from CT reports

Parameter	All (*n* = 2,923)	PE (*n* = 867)	No PE (*n* = 2,056)	*p*-value
Age (years)*	66 ± 17	67 ± 16	66 ± 17	0.20
Sex				
Women	1,465 (50.1)	427 (49.3)	1,038 (50.5)	0.29
Men	1,458 (49.9)	440 (50.7)	1,018 (49.5)	0.29
Presence of PE				
No presence of PE	2,027 (69.3)	0 (0)	2,027 (98.6)	
Presence of PE	810 (27.7)	810 (93.4)	0 (0)	
Suspicion of PE	57 (2)	57 (6.6)	0 (0)	
Not assessable	26 (0.9)	0 (0)	26 (1.3)	
Missing field	1 (0)	0 (0)	1 (0)	
Invalid value	2 (0.1)	0 (0)	2 (0.1)	
PE localisation				
MPA	377 (12.9)	377 (43.5)	0 (0)	
Lobar	229 (7.8)	229 (26.4)	0 (0)	
Segmental	173 (5.9)	173 (20)	0 (0)	
Subsegmental	65 (2.2)	65 (7.5)	0 (0)	
Unclassified^†^	23 (0.8)	23 (2.7)	0 (0)	
Missing field	97 (3.3)	97 (11.2)	0 (0)	
Invalid value	75 (2.6)	71 (8.2)	4 (0.2)	
PE severity levels^‡^				
No PE	2,027 (69.3)	0 (0)	2,027 (98.6)	
Low (< 25%)	366 (12.5)	366 (42.2)	0 (0)	
Intermediate (25–50%)	143 (4.9)	143 (16.5)	0 (0)	
High (> 50%)	286 (9.8)	286 (33.0)	0 (0)	
Error (out-of-range)^§^	70 (2.4)	43 (5.0)	27 (1.3)	
Invalid value	34 (1.2)	29 (3.3)	5 (0.2)	
Signs of RHS				
RV/LV ratio < 1	2,272 (77.7)	451 (52)	1,821 (88.6)	< 0.001
RV/LV ratio ≥ 1	639 (21.9)	414 (47.8)	225 (10.9)	< 0.001
Missing field	10 (0.3)	0 (0)	10 (0.5)	
Invalid value	2 (0.1)	2 (0.2)	0 (0)	
Perfusion deficit				
None	752 (25.7)	73 (8.4)	679 (33)	< 0.001
< 25%	301 (10.3)	162 (18.7)	139 (6.8)	< 0.001
≥ 25%	207 (7.1)	192 (22.1)	15 (0.7)	< 0.001
Missing field	32 (1.1)	13 (1.5)	19 (0.9)	
Invalid value	8 (0.3)	7 (0.8)	1 (0)	
Attenuation of pulmonary trunk				
Assessment performed	2,922 (100)	867 (100)	2,055 (100)	
Attenuation (HU)*	339 ± 155	345 ± 143	336 ± 160	0.14
Missing field	2 (0.1)	2 (0.2)	0 (0)	
ECG synchronisation				
No ECG sync	2,658 (90.9)	778 (89.7)	1,880 (91.4)	0.51
ECG sync	263 (9)	88 (10.1)	175 (8.5)	0.51
Missing field	2 (0.1)	1 (0.1)	1 (0)	
Artefacts				
No artefacts	1,217 (41.6)	378 (43.6)	839 (40.8)	< 0.001
Minimal artefacts	1,042 (35.6)	321 (37)	721 (35.1)	< 0.001
Mild artefacts	389 (13.3)	102 (11.8)	287 (14)	< 0.001
Moderate artefacts	191 (6.5)	41 (4.7)	150 (7.3)	< 0.001
Severe artefacts	72 (2.5)	21 (2.4)	51 (2.5)	< 0.001
Non-diagnostic	8 (0.3)	1 (0.1)	7 (0.3)	< 0.001
Missing field	2 (0.1)	2 (0.2)	0 (0)	
Invalid value	1 (0)	1 (0.1)	0 (0)	

Unless otherwise specified, data are frequencies, with percentages in parentheses

*LV* Left ventricle, *MPA* Main pulmonary arteries, *RHS* Right heart strain, *RV* Right ventricle

* Data are mean ± standard deviation

^†^ Denotes reports that were marked positive for pulmonary embolism (PE) at the report level but for which no specific embolus location was documented in the PE module

^‡^ Denotes severity levels of PE according to a semiquantitative clot burden score

^§^ Indicates out-of-range values in the PE severity variable, according to the clot burden score (valid range: 0–40)

### Human baseline performance and variability

Across all eight radiologists and 40 922 entries, pooled item-level performance *versus* the reference was high (precision, 92.1% [95% CI, 89.9–92.8]; recall, 91.8% [95% CI, 90.3–93.1]; F1, 0.92 [95% CI, 0.90–0.93]). Item-level accuracy was 99.5% (95% CI, 99.3–99.7). Inter-reader agreement across the 120 overlapping reports was characterised by a Fleiss’ κ of 0.985 (pairwise κ range, 0.968–0.998). Intra-reader reproducibility on duplicate reports yielded a pooled Cohen’s κ of 0.97 (reader-wise range, 0.93–1.00). Radiologists required a cumulative 32.8 h to label the entire cohort (mean, 43.4 s per report).

### Rule-based extractor performance

Using the corrected labels as reference, the RBE alone achieved high item-level performance (precision, 83.1% [95% CI, 83.1–84.3]; recall, 78.4% [95% CI, 77.5–80.1]; F1, 0.81 [95% CI, 0.80–0.82]). Item-level accuracy was 98.5% (95% CI, 98.3–98.7). Errors occurred predominantly in fields characterised by variable phrasing, such as *post hoc* free-text additions and alternative wording deviating from the PE template (Fig. 2).

### LLM-only labelling performance

**Fig. 3:**
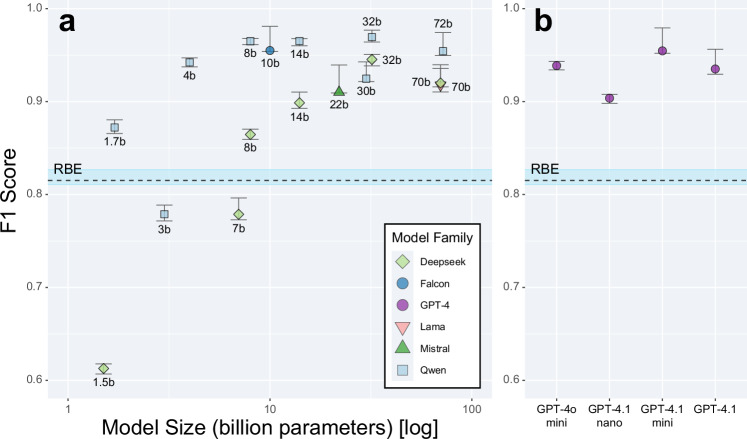
Model performance by scale. F1 score is plotted against model size for open-weight (**a**) and proprietary large language models (**b**). A horizontal dashed line denotes the F1 score of the rule-based extractor (RBE). Error bars denote 95% confidence intervals. Model sizes for GPT-4 variants are not publicly disclosed

Among the 22 LLMs, GPT-4.1-mini and Falcon3-10b achieved the highest item-level performance, with F1 scores of 0.98 (95% CI, 0.97–0.98) and 0.98 (95% CI, 0.97–0.98), respectively. Their F1 scores did not show evidence of a difference (*p* = 0.70), and both exceeded the RBE (*p* < 0.001 for each comparison). Model performance by parameter scale is shown in Fig. 3, and detailed performance metrics for all models are provided in Table 2.

**Table 2:** Labelling performance of radiologists, RBE and LLM pipelines

Parameter	Precision (%)	Recall (%)	Accuracy (%)	F1 score
Radiologists (pooled)	92.1 (89.9, 92.8)	91.8 (90.3, 93.1)	99.5 (99.3, 99.7)	0.92 (0.90, 0.93)
RBE	83.1 (83.1, 84.3)	78.4 (77.5, 80.1)	98.5 (98.3, 98.7)	0.81 (0.80, 0.82)
Open-weight LLM				
Falcon3-10b	97.8 (97.0, 98.4)	97.7 (97.0, 98.2)	99.5 (99.4, 99.6)	0.98 (0.97, 0.98)
Mistral-small-22b	92.7 (91.7, 93.7)	96.9 (96.2, 97.5)	98.6 (98.5, 98.8)	0.94 (0.94, 0.95)
Llama3.3-70b	90.6 (89.4, 91.7)	98.2 (97.7, 98.7)	98.8 (98.6, 99.0)	0.93 (0.92, 0.94)
Qwen2.5-3b	83.9 (82.7, 85.1)	86.3 (85.3, 87.2)	94.7 (94.4, 95.1)	0.83 (0.81, 0.84)
Qwen2.5-72b	95.2 (94.3, 96.1)	99.1 (98.7, 99.4)	99.4 (99.2, 99.5)	0.97 (0.96, 0.98)
Qwen3-0.6b	84.0 (82.9, 85.3)	89.7 (88.6, 90.7)	94.2 (93.9, 94.5)	0.86 (0.85, 0.87)
Qwen3-1.7b	86.3 (85.0, 87.5)	90.7 (90.0, 91.4)	96.2 (95.8, 96.5)	0.87 (0.86, 0.88)
Qwen3-4b	94.5 (93.3, 95.5)	93.9 (93.1, 94.7)	98.6 (98.5, 98.8)	0.93 (0.92, 0.94)
Qwen3-8b	96.1 (95.3, 97.0)	96.2 (95.4, 96.9)	99.1 (99.0, 99.2)	0.96 (0.95, 0.97)
Qwen3-14b	94.4 (93.2, 95.5)	98.5 (98.1, 98.9)	99.4 (99.3, 99.5)	0.96 (0.95, 0.97)
Qwen3-30b	95.4 (94.3, 96.5)	93.7 (92.6, 94.8)	98.8 (98.6, 98.9)	0.94 (0.93, 0.95)
Qwen3-32b	95.5 (94.5, 96.5)	98.2 (97.8, 98.6)	99.4 (99.3, 99.5)	0.97 (0.96, 0.97)
Deepseek-R1-1.5b	63.6 (61.8, 65.2)	75.1 (73.7, 76.3)	85.2 (84.5, 85.9)	0.65 (0.64, 0.66)
Deepseek-R1-7b	79.6 (78.5, 80.5)	89.6 (88.6, 90.6)	93.1 (92.6, 93.5)	0.81 (0.80, 0.82)
Deepseek-R1-8b	87.8 (86.9, 88.7)	96.7 (96.2, 97.2)	97.0 (96.7, 97.2)	0.90 (0.90, 0.91)
Deepseek-R1-14b	92.4 (91.2, 93.5)	97.7 (97.2, 98.2)	98.6 (98.4, 98.8)	0.95 (0.94, 0.95)
Deepseek-R1-32b	95.0 (93.9, 96.1)	98.7 (98.3, 99.1)	99.4 (99.3, 99.5)	0.97 (0.96, 0.97)
Deepseek-R1-70b	93.6 (92.5, 94.7)	98.4 (98.0, 98.8)	99.1 (99.0, 99.3)	0.96 (0.95, 0.96)
Proprietary LLM				
GPT 4o-mini	94.5 (93.6, 95.4)	97.8 (97.4, 98.3)	98.9 (98.8, 99.1)	0.96 (0.95, 0.96)
GPT 4.1-nano	89.5 (88.5, 90.4)	96.6 (96.0, 97.2)	97.8 (97.6, 98.0)	0.92 (0.91, 0.93)
GPT 4.1-mini	96.2 (95.3, 97.1)	99.2 (98.8, 99.5)	99.4 (99.3, 99.6)	0.98 (0.97, 0.98)
GPT 4.1	96.1 (94.9, 97.2)	99.6 (99.4, 99.8)	99.5 (99.3, 99.6)	0.98 (0.97, 0.98)

Unless otherwise specified, data are percentages, with 95% CIs in parentheses. Accuracy denotes the proportion of individual template fields correctly extracted. Performance metrics are based on consolidated ground truth annotations derived from rule-based extractions provided by the two attending radiologists

*LLM* Large language models, *RBE* Rule-based extractor

**Fig. 4:**
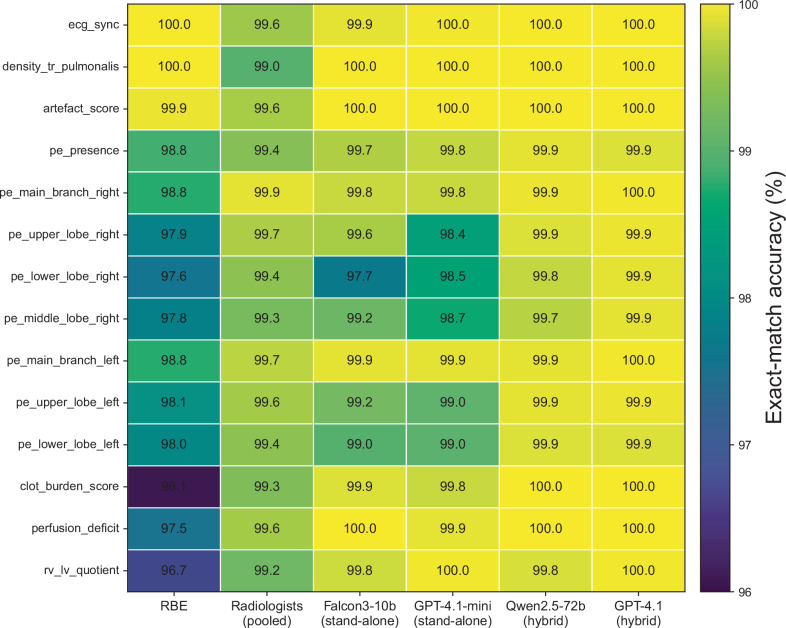
Field-level exact-match accuracy across all 14 template variables. Heatmap showing field-level exact-match accuracy for the rule-based extractor (RBE), pooled radiologists, the two best-performing standalone models, and the best-performing open-weight and proprietary hybrid models selected according to pooled item-level performance. The heatmap complements the pooled summary metrics in Table 2 by visualising parameter-specific heterogeneity across the structured pulmonary embolism CT reporting schema. Exact-match accuracy denotes the proportion of field entries identical to the reference standard for a given template variable

Figure 4 illustrates the heterogeneity of the 14 template variables at the field level. The largest differences in exact-match accuracy were observed for the embolus localisation fields, the perfusion deficit field, the clot burden score field, and the RV/LV quotient field. In contrast, the ECG synchronisation field, the pulmonary trunk attenuation field, and the artefact score field were extracted with near-perfect accuracy by all methods. The RBE demonstrated the lowest field-level accuracy for the clot burden score (96.1%), the RV/LV quotient (96.7%), the perfusion deficit (97.5%), and several lobar localisation fields (97.6–98.1%). In contrast, the performance of pooled radiologists and the best standalone and hybrid models was uniformly high across all fields, indicating that the remaining performance gap was driven by a small subset of more variable PE-related labels.

### Hybrid RBE–LLM performance

**Fig. 5:**
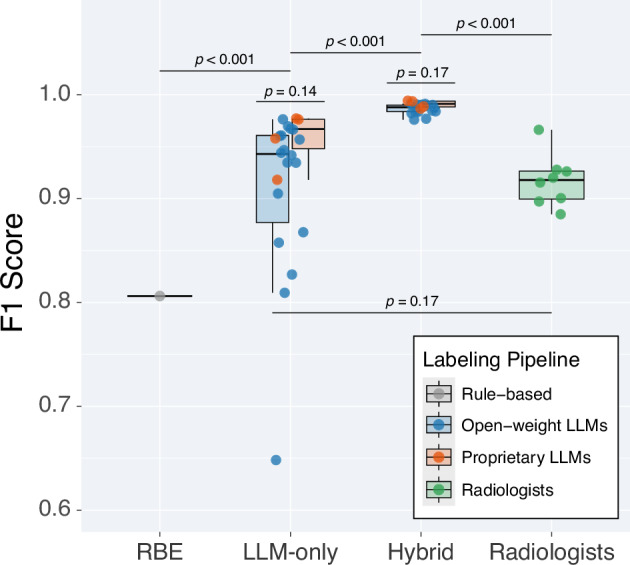
Performance of three labelling pipelines. F1 scores are shown for the rule-based extractor (RBE), standalone large language-model (LLM) extraction, and a hybrid workflow in which RBE failures are salvaged by the corresponding LLM. Boxes depict the median and interquartile range, whiskers the 5th–95th percentiles; jittered dots represent individual models. Horizontal brackets report two-sided Wilcoxon signed-rank *p*-values for the paired comparison between standalone LLM and hybrid approach and two-sided Mann–Whitney *U* values for the unpaired comparison between open-weight and proprietary models within the same pipeline

Overall performance of the rule-based, LLM-only, and hybrid pipelines is shown in Table 3 and Fig. 5. For the 647 RBE failures in the reference standard, median salvage across open-weight models was 88.1% (interquartile range (IQR), 84.0–89.9%) and 91.9% (IQR, 88.5–94.8%) across proprietary models without evidence of a difference between both model groups (*p* = 0.14). Using the hybrid workflow that applied RBE first and routed only failures to an LLM increased the median item-level F1 from 0.81 (IQR, 0.81–0.81) for RBE alone to 0.99 (IQR, 0.98–0.99) across models (*p* < 0.001).

**Table 3:** Labelling performance and time across readers and pipelines

Parameter	*n*	F1 score	Accuracy (%)	Salvage (%)	Duration (h)
Radiologists	8	0.92 (0.90, 0.93)	99.5 (99.4, 99.6)		32.2 (30.5, 36.8)
RBE	1	0.81 (0.81, 0.81)	98.5 (98.5, 98.5)		≈ 0
LLM-only pipeline	22	0.95 (0.91, 0.97)	98.8 (97.2, 99.4)		6.7 (5.2, 16.3)
Open-weight LLM	18	0.94 (0.88, 0.96)	98.7 (96.4, 99.3)		8.1 (5.7, 19.5)
Proprietary LLM	4	0.97 (0.95, 0.98)	99.2 (98.7, 99.4)		5.2 (5.1, 5.5)
*p*-value		0.17	0.23		0.12
Hybrid LLM pipeline	22	0.99 (0.98, 0.99)	99.8 (99.8, 99.9)	88.2 (84.8, 90.9)	1.0 (0.8, 2.4)
Open-weight LLM	18	0.99 (0.98, 0.99)	99.8 (99.8, 99.9)	88.1 (84.0, 89.9)	1.2 (0.8, 2.8)
Proprietary LLM	4	0.99 (0.99, 0.99)	99.9 (99.8, 99.9)	91.9 (88.5, 94.8)	0.8 (0.8, 0.8)
*p*-value		0.14	0.12	0.12	0.14
Workflow comparison (*p*-values)					
Radiologists *vs* LLM-only		0.17	0.001		0.001
Radiologists *vs* hybrid		< 0.001	< 0.001		< 0.001
LLM-only *vs* hybrid		< 0.001	< 0.001		< 0.001

Unless otherwise specified, data are presented as median, with interquartile ranges in parentheses. Duration reflects the projected time each model or reader would require to process the full study cohort. For radiologists, time refers to the median projected cohort reading time if a single reader labelled all 2,923 reports. “Salvage” denotes the proportion of template fields in which the rule-based extractor (RBE) failed but were subsequently recovered correctly by the large language models (LLM). “Hybrid” refers to a staged pipeline in which the LLM is invoked only for RBE failures. RBE execution was performed locally, and the negligible computational time was set to ≈ 0

Residual hybrid errors were rare and confined to a small number of PE-related fields. The most common failure occurred when both the adjudicated reference standard and the RBE retained a field as invalid, but the LLM nevertheless selected a specific schema value rather than preserving non-resolvability. Additional residual errors mainly involved ambiguous embolus localisation and qualified or semiquantitative source expressions, particularly regarding thrombus burden, RV/LV quotient and perfusion deficit. Representative examples are provided in Appendix E4.

Compared with running LLMs on all reports, the hybrid workflow reduced the number of LLM calls by 85.6%, as only reports with RBE failures required LLM salvage. Across all hybrid configurations, the median cohort labelling time was 1.0 h (IQR, 0.8–2.4 h). For radiologists, the projected median single-reader cohort time was 32.2 h (IQR, 30.5–36.8 h), while the observed cumulative reading time across all eight readers was 32.8 h (mean, 40.4 s per report). In comparison, standalone open-weight and proprietary LLMs showed median cohort runtimes of 8.1 h (IQR, 5.7–19.5 h) and 5.2 h (IQR, 5.1–5.5 h), respectively. Several individual LLMs achieved F1 scores equal to or higher than the pooled radiologist performance (F1, 0.92; 95% CI, 0.90–0.93). Among the open-weight models, this included Falcon3-10b, Qwen3-14b, Qwen3-30b, Qwen3-32b and Qwen3-8b. Among the proprietary models, GPT-4.1-mini reached a comparable F1 score (Table 2).

## Discussion

Open-weight large language models (LLMs) have been proposed as an alternative to proprietary systems that preserve privacy for clinical language processing. In this study, selected open-weight models achieved an F1 score of up to 0.98, which is comparable to that of proprietary GPT-4 variants (up to 0.98) and exceeds that of a rules-based extractor (RBE; F1 score of 0.81; *p* < 0.001). An eight-radiologist baseline demonstrated high levels of labelling agreement but required 32.8 h in total for the annotation of the entire cohort (F1 = 0.92). This was superseded in terms of report-level accuracy by a rules-first workflow that entailed targeted adjudication of RBE failures. The salvage rate for rule-failed fields was similar for open-weight (88.1%) and proprietary models (91.9%; *p* = 0.12). Standalone median annotation times were 8.1 h for open-weight LLMs and 5.2 h for proprietary LLMs, and routing only RBE failures to an LLM yielded near-perfect labelling (99.8% accuracy; F1 = 0.99) while reducing cohort time to 1.0 h (*p* < 0.001).

When interpreting these findings, it is important to consider the nature of the target task. This study evaluated schema-constrained extraction from finalised structured report text rather than diagnostic reasoning or image-based clinical truth adjudication. All compared systems operated within the same constrained label space defined by the reporting template. Accordingly, the relevant comparison was not image-level diagnosis, but the fidelity with which structured report content was transferred into the predefined schema. A random audit of 400 initially accepted RBE-valid fields identified no confirmed silent parsing errors, which further supports the robustness of the report-derived reference standard for this extraction task.

Hybrid architectures that combine deterministic components with machine-learning models have been proposed in clinical decision support and information extraction to balance precision, computational cost, and maintainability [14, 15]. Our results suggest that the most effective information retrieval strategy depends on the degree of report standardisation and on how structured content is handled in routine workflows. For highly rigid templates with fixed value sets and little linguistic variability, compact rule sets can provide near-perfect performance with minimal runtime. In our study, the PE reporting template itself is highly structured and itemised, but the final reports behave as structured documents because the template output is copied into the RIS word processor and can be modified *post hoc*. Minor deviations introduced during this step—such as edited phrasing, free-text additions, or partially altered lists—can break exact-match rules, making purely rule-based extraction less reliable. In this setting, LLMs can compensate for template deviations and recover a large proportion of rule-failed fields. For predominantly free-text reports, in which linguistic variability and implicit information dominate, LLM-based extraction is likely to be the more suitable primary option. Our structured PE reporting environment, therefore, lies between fully structured and fully free-text formats and illustrates how a rules-first, LLM-salvage strategy can combine the robustness of structured templates with the flexibility of modern language models.

A further consideration is the balance between engineering effort and runtime efficiency. Developing and maintaining a robust RBE requires initial scripting effort and detailed knowledge of the reporting template, and rules must be updated when the template or coding scheme changes. Once implemented, however, rules are transparent, easy to audit, and computationally efficient. LLMs, in contrast, can often be integrated with comparatively little task-specific engineering, typically *via* prompt design rather than field-specific code, lowering the entry barrier for institutions without dedicated natural language processing expertise [16, 17]. This comes at the cost of higher computational demand and dependence on model provisioning. Our results indicate that institutions with established structured reporting can use rules as a fast and interpretable backbone while reserving LLMs for the smaller subset of cases in which rules fail or language falls outside the predefined schema.

Our findings also have implications for deployment choices. In this template-constrained task, open-weight LLMs performed similarly to proprietary GPT-4 variants. This suggests that on-premises inference could be a viable option for institutions that prioritise data governance and local control, and that have the necessary high-end GPU infrastructure and technical support [18, 19]. However, the local setup used in this study relied on a 48 GB professional GPU and should therefore not be interpreted as representative of typical radiology department baseline resources. Hardware of this class is associated with substantial cost, typically in the upper four- to low five-figure US-dollar range depending on vendor, market, and configuration. At the same time, cloud-hosted models may simplify maintenance and provide competitive performance without local hardware investment. Because all models were accessed through the same structured-output interface, the choice between open-weight and proprietary systems can be adapted over time without altering the analytic pipeline, which is advantageous in a rapidly evolving model landscape.

This study has limitations. First, our analysis was limited to structured CT pulmonary angiography reports from a single institutional network, all based on one reporting template. Performance in free-text reports or reports based on different templates may differ, so multi-centre validation is therefore needed. Second, we only evaluated the extraction task; downstream clinical consequences of residual labelling errors for outcome studies, risk stratification or quality metrics were not assessed. A separate resource-focused analysis examined the time, cost and carbon footprint of these pipelines in more detail. Third, the reference standard remained report-derived rather than image-derived. Although the adjudicated RBE table was supported by a random audit of 400 initially accepted RBE-valid fields with no confirmed silent RBE errors, a very small residual error rate cannot be excluded. Accordingly, our analyses address the accuracy of extraction from finalised structured report text rather than diagnostic accuracy relative to source images. Finally, although we included a broad range of open-weight and proprietary LLMs, model families and versions evolve rapidly, and future models may perform differently.

In conclusion, in this schema-constrained extraction task, both open-weight and proprietary large language models (LLMs) matched or exceeded the performance of rule-based extraction, while the hybrid rules-first workflow achieved the highest overall performance and matched or exceeded a pooled radiologist label-transfer baseline. A targeted rules-first LLM workflow appears to be a practical and scalable approach for high-fidelity labelling of finalised structured pulmonary embolism CT reports.

## Supplementary information

**Additional File :** **Appendix E1:** Technical specifications of large language models. **Appendix E2:** Characteristics of labelled embolus locations. **Appendix E3:** Audit validation workflow and item distribution. **Appendix E4:** Residual hybrid failure cases: representative examples.

## Data Availability

Due to data protection regulations and institutional policies, the full set of original clinical reports cannot be shared publicly. Access to pseudonymised label tables may be considered on reasonable request and subject to data-sharing agreements with the University Hospital Heidelberg.

## Code availability

The code for the rule-based and LLM labelling pipelines, together with configuration files and example schemas, is available in a public GitHub repository.
