# Supplementary material for: Who labels best? Radiologists, rules, or large language models for CT reports on pulmonary embolism
Source: Eur Radiol Exp. 2026 May 27;10:74. doi: 10.1186/s41747-026-00738-7 (PMC13216375; doi:10.1186/s41747-026-00738-7)
Supplement: Supplementary file 1 — Additional File : Appendix E1: Technical specifications of large language models. Appendix E2: Characteristics of labelled embolus locations. Appendix E3: Audit validation workflow and item distribution. Appendix E4: Residual hybrid failure cases: representative examples. [file 41747_2026_738_MOESM1_ESM.pdf]

Who labels best? Radiologists, rules, or large language models for CT reports on pulmonary embolism
ELECTRONIC SUPPLEMENTARY MATERIAL

Appendix E1: Technical specifications of large language models

Parameter	Provider	Release Date	License	Context Length	Source
Open-weight LLM					
Llama 3.3-70b	Meta	2024	Llama Community	131,072	Ollama
Mistral-small-22b	Mistral AI	2024	Mistral AI Research	131,072	Ollama
Falcon3-10b	TII / MegaSys	2024	Falcon 3 TII	32,768	Ollama
Qwen2.5-72b	Alibaba Group	2024	Apache 2.0	32,768	Ollama
Qwen2.5-3b	Alibaba Group	2024	Apache 2.0	32,768	Ollama
Qwen3-0.6b	Alibaba Group	2025	Apache 2.0	40,960	Ollama
Qwen3-1.7b	Alibaba Group	2025	Apache 2.0	40,960	Ollama
Qwen3-4b	Alibaba Group	2025	Apache 2.0	40,960	Ollama
Qwen3-8b	Alibaba Group	2025	Apache 2.0	40,960	Ollama
Qwen3-14b	Alibaba Group	2025	Apache 2.0	40,960	Ollama
Qwen3-30b	Alibaba Group	2025	Apache 2.0	40,960	Ollama
Qwen3-32b	Alibaba Group	2025	Apache 2.0	40,960	Ollama
Deepseek-R1-1.5b	DeepSeek	2025	MIT	131,072	Ollama
Deepseek-R1-7b	DeepSeek	2025	MIT	131,072	Ollama
Deepseek-R1-8b	DeepSeek	2025	MIT	131,072	Ollama
Deepseek-R1-14b	DeepSeek	2025	MIT	131,072	Ollama
Deepseek-R1-32b	DeepSeek	2025	MIT	131,072	Ollama
Deepseek-R1-70b	DeepSeek	2025	MIT	131,072	Ollama
Proprietary LLM					
GPT-4o-mini	OpenAI	2024	Proprietary	~ 128,000	OpenAI
GPT-4.1-nano	OpenAI	2025	Proprietary	~ 1,000,000	OpenAI
GPT-4.1-mini	OpenAI	2025	Proprietary	~ 1,000,000	OpenAI
GPT-4.1	OpenAI	2025	Proprietary	~ 1,000,000	OpenAI

Context length refers to the maximum tokens processable in a single prompt-response pair. Sources included model cards, press releases, and official documentation. LLM = large language models.

Appendix E2: Characteristics of labelled embolus locations

Parameter	No. of PEs
Successful PE extractions	2492 (100)
MPA right	337 (13.5)
Total occlusion	31 (1.2)
Partial occlusion	306 (12.3)
MPA left	255 (10.2)
Total occlusion	19 (0.8)
Partial occlusion	236 (9.5)
Right upper lobe	357 (14.3)
Total occlusion of lobar artery	48 (1.9)
Partial occlusion of lobar artery	162 (6.5)
Segmental PE	108 (4.3)
Subsegmental PE	39 (1.6)
Middle lobe	310 (12.4)
Total occlusion of lobar artery	54 (2.2)
Partial occlusion of lobar artery	140 (5.6)
Segmental PE	80 (3.2)
Subsegmental PE	36 (1.4)
Right lower lobe	460 (18.5)
Total occlusion of lobar artery	50 (2)
Partial occlusion of lobar artery	179 (7.2)
Segmental PE	167 (6.7)
Subsegmental PE	64 (2.6)
Left upper lobe	340 (13.6)
Total occlusion of lobar artery	29 (1.2)
Partial occlusion of lobar artery	137 (5.5)
Segmental PE	122 (4.9)
Subsegmental PE	52 (2.1)
Left lower lobe	433 (17.4)
Total occlusion of lobar artery	26 (1)
Partial occlusion of lobar artery	173 (6.9)
Segmental PE	164 (6.6)
Subsegmental PE	70 (2.8)

Data in parentheses are percentages. Level of pulmonary embolism (PE) is provided on a per-level basis. MPA = main pulmonary artery.

Appendix E3: Audit validation workflow and item distribution

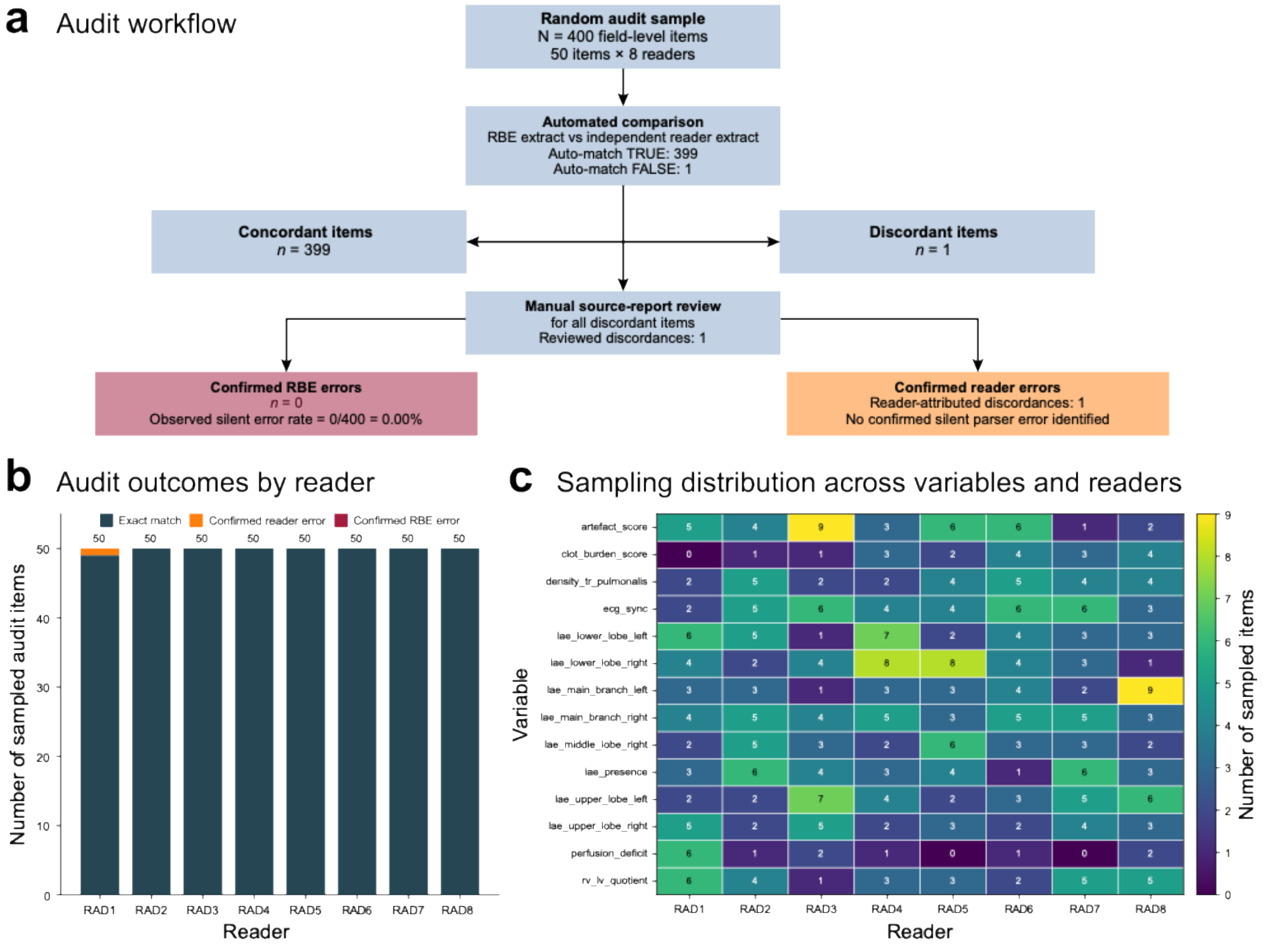

Audit validation workflow and item distribution. (**a**) Two-stage audit of initially accepted rule-based extractor (RBE)-valid fields. A random sample of 400 field-level items was drawn from reports with fully schema-valid, rule-based extraction results. Fifty items were sampled per reader, with sampling restricted to one item per report. An automated comparison of the RBE extract and the independently recorded reader extract yielded 399 concordant items and one discordant item. All discordant items underwent manual review of the source report. The single discordance was attributed to the reader's extraction process rather than the RBE itself, resulting in zero confirmed silent RBE errors among the 400 audited items. (**b**) Audit outcomes by reader. Each of the eight radiologists contributed 50 sampled items. All readers showed complete or near-complete concordance with the RBE. The only discordant item occurred in one reader subset and was resolved as a reader error upon manual review. (**c**) A heatmap showing the distribution of sampled audit items across the 14 template variables and eight readers demonstrates broad field coverage rather than concentration on a small subset of labels.

Appendix E4: Residual hybrid failure cases: representative examples

Variable	Error type	Report excerpt	Explanation
clot_burden_score	LLM forced schema value on invalid source	"Heidelberg Clot Burden Score …: =#Right+2.5"	The structured field contained a malformed non-schema clot burden entry. RBE and adjudicated ground truth therefore correctly retained the field as invalid, whereas the LLM still converted it into a numeric schema value.
artefact_score	LLM forced schema value on invalid source	"Artefact score (0–5): 3–4 (marked respiratory artefacts)"	The source used a range rather than one permitted discrete schema value. RBE and ground truth remained invalid, but the LLM collapsed the range to a single score.
lae_presence	Ambiguous embolus localisation / non-resolvability	"Peripheral arteries not sufficiently assessable. No central pulmonary embolism."	The source explicitly excluded central PE while also stating limited peripheral assessability. This did not support a clean positive schema mapping, but the LLM nevertheless assigned a concrete PE label.
lae_upper_lobe_right	Ambiguous embolus localisation	"Right upper lobe: occluded at segmental artery level … continuation of thrombus into segment II artery … subsegmental occlusion in ventral segment III."	The report mixed segmental and subsegmental localisation within the same lobe. The LLM appears to have over-specified the embolus level instead of preserving the adjudicated schema mapping.
rv_lv_quotient	Numeric / wording irregularity	"RV/LV ratio: not elevated"	The RV/LV field was expressed verbally rather than as a clean schema token. RBE left it invalid, whereas the LLM mapped the wording to a concrete categorical value.
perfusion_deficit	Qualified or semi-quantitative expression	"Perfusion deficits (dual-energy CT): =25% (however difficult to assess in severe emphysema)"	The source combined a threshold-like value with an explicit qualification about limited assessability. The LLM simplified this qualified statement to a concrete schema label.
lae_lower_lobe_left	Ambiguous embolus localisation	"Left lower lobe: partially occlusive … saddle embolus … partly complete, partly incomplete occlusion."	The report described mixed downstream occlusion patterns in the context of a saddle embolus. The LLM appears to have over-committed to a single localisation/severity label relative to the adjudicated schema mapping.

Representative residual failure modes of the hybrid RBE–LLM workflow. This table shows examples of residual hybrid errors, i.e. cases where the rule-based extractor (RBE) failed and the large language model (LLM) did not recover the adjudicated reference-standard label. The most common failure occurred when the reference standard and the RBE correctly identified a field as invalid, but the LLM selected a specific schema value anyway. Additional residual errors mainly involved ambiguous embolus localisation and qualified or semiquantitative source expressions.
